# Isolated Cortical Vein Thrombosis of the Vein of Trolard Mimicking a Brain Tumor in Pregnancy: A Case Report

**DOI:** 10.7759/cureus.102169

**Published:** 2026-01-23

**Authors:** Ali O Al‎zahr‎ani, Abdulmajeed M Alwzynani, Feras M Aldhubayi, Amal M Alahmari, Anas E Ahmed

**Affiliations:** 1 College of Medicine, University of Jeddah, Jeddah, SAU; 2 College of Medicine, Umm Al-Qura University, Makkah, SAU; 3 College of Pharmacy, Qassim University, Buraydah, SAU; 4 College of Medicine, King Khalid University, Abha, SAU; 5 Community Medicine, Jazan University, Jazan, SAU

**Keywords:** cerebral venous thrombosis, cortical vein thrombosis, hypercoagulable state, magnetic resonance imaging, pregnancy, tumor mimicker, vein of trolard, venous infarction

## Abstract

Cortical vein thrombosis is a rare and frequently underrecognized cause of acute focal neurological deficits, particularly when it presents with imaging features that mimic intracranial tumors. We report the case of a pregnant woman who presented with progressive headache and focal neurological deficits, in whom MRI revealed a left parietal cortical-subcortical lesion with surrounding edema and mass effect, initially suggestive of a space-occupying lesion. Detailed evaluation using diffusion-weighted and susceptibility-sensitive sequences demonstrated findings consistent with isolated thrombosis of the vein of Trolard, resulting in venous infarction. Pregnancy-related hypercoagulability was identified as the most likely predisposing factor. The patient was managed conservatively with therapeutic low-molecular-weight heparin and supportive measures following multidisciplinary discussion, leading to rapid clinical improvement and favorable maternal and fetal outcomes. Follow-up imaging confirmed resolution of edema and partial venous recanalization, with no evidence of an underlying neoplastic process. This case highlights the diagnostic challenges posed by isolated cortical vein thrombosis, particularly in pregnant patients, and emphasizes the critical role of advanced MRI techniques and high clinical suspicion in differentiating venous infarction from tumor-like lesions. Early recognition and timely anticoagulation are essential to prevent unnecessary interventions and ensure optimal outcomes.

## Introduction

Cortical vein thrombosis (CVT) is an uncommon and often underdiagnosed subtype of cerebral venous thrombosis, accounting for a small proportion of cerebrovascular events [1,2]. Unlike dural sinus thrombosis, isolated involvement of cortical veins presents a significant diagnostic challenge due to its nonspecific clinical manifestations and highly variable radiological appearance [2,3]. Patients may present with headache, focal neurological deficits, seizures, or altered mental status, and imaging findings can closely resemble intracranial neoplasms, infections, or inflammatory lesions [1-3]. The vein of Trolard, a major superficial anastomotic vein connecting the superficial middle cerebral vein to the superior sagittal sinus, is a rare site of isolated thrombosis, and its involvement may result in focal venous infarction with mass effect, further contributing to diagnostic confusion [2-4].

Pregnancy is a well-recognized hypercoagulable state due to physiological changes in coagulation factors, venous stasis, and endothelial alterations, which together increase the risk of thromboembolic events, including cerebral venous thrombosis [1,4]. However, isolated CVT during pregnancy remains exceedingly rare, with limited cases reported in the literature [1-8]. Early recognition is crucial, as prompt anticoagulation can lead to favorable maternal and fetal outcomes, whereas delayed or missed diagnosis may result in significant morbidity [2,3]. This case highlights the importance of maintaining a high index of suspicion for CVT in pregnant patients presenting with focal neurological deficits and tumor-like brain lesions on imaging, underscoring the critical role of advanced MRI sequences in establishing an accurate diagnosis.

## Case presentation

A 28-year-old primigravida woman at approximately 24 weeks of gestation presented to the emergency department with a five-day history of progressively worsening headache. The headache was described as constant, dull to throbbing in character, predominantly involving the left parietal region, and partially relieved with acetaminophen. It was associated with intermittent nausea and two episodes of non-projectile vomiting. One day before the presentation, she developed new-onset right-sided weakness and difficulty in word finding, which prompted urgent medical evaluation. There was no history of fever, trauma, loss of consciousness, seizures, visual disturbances, or prior similar episodes. She denied symptoms suggestive of preeclampsia, including visual blurring, epigastric pain, or limb edema. Her antenatal course until presentation had been unremarkable, with regular follow-up and no reported complications.

Her past medical history was notable only for iron-deficiency anemia treated with oral iron supplementation. She had no personal or family history of thromboembolic disease, stroke, migraine, malignancy, autoimmune disorders, or known thrombophilia. She was not taking oral contraceptives or other medications apart from prenatal vitamins and iron supplements. There was no history of smoking, alcohol consumption, or illicit drug use. Obstetric history revealed a spontaneous conception, and routine antenatal ultrasounds had demonstrated a normally developing fetus. There was no recent history of infection, dehydration, prolonged immobilization, or surgical procedures.

On physical examination, she was alert but appeared distressed due to a headache. Vital signs were within normal limits, with blood pressure of 112/70 mmHg, pulse rate of 86 beats per minute, respiratory rate of 16 breaths per minute, and she was afebrile. General examination revealed mild pallor but no jaundice, cyanosis, or peripheral edema. Obstetric examination was unremarkable, with uterine size appropriate for gestational age and normal fetal heart tones. Neurological examination demonstrated mild expressive aphasia and right-sided hemiparesis with Medical Research Council grade 4/5 power in both the upper and lower limbs. Deep tendon reflexes were brisk on the right side, with an equivocal plantar response. Sensory examination revealed no objective deficits. Cranial nerve examination was normal, and there were no signs of meningeal irritation. Fundoscopic examination did not reveal papilledema.

Initial laboratory investigations showed hemoglobin of 10.2 g/dL with microcytic indices, consistent with known iron-deficiency anemia. White blood cell count and platelet count were within normal ranges. Serum electrolytes, renal function tests, and liver function tests were unremarkable. Coagulation profile, including prothrombin time and activated partial thromboplastin time, was within normal limits. Urinalysis showed no proteinuria. Inflammatory markers were not elevated. Given her pregnancy and focal neurological deficits, neuroimaging was prioritized (Table 1).

**Table 1:** Summary of laboratory investigations at presentation and during diagnostic workup. The table summarizes the hematological, biochemical, immunological, and urinary laboratory findings obtained during the patient’s initial evaluation. Reference ranges correspond to standard adult values. HPF = high-power field

Laboratory test	Patient value	Unit	Reference range
Hemoglobin	10.2	g/dL	12.0–15.5
Hematocrit	31.5	%	36–46
Red blood cell count	3.8	×10⁶/µL	4.0–5.2
Mean corpuscular volume	78	fL	80–96
Mean corpuscular hemoglobin	26	pg	27–33
Mean corpuscular hemoglobin concentration	32	g/dL	32–36
Red cell distribution width	15.2	%	11.5–14.5
White blood cell count	8.1	×10³/µL	4.0–11.0
Neutrophils	62	%	40–75
Lymphocytes	30	%	20–45
Monocytes	6	%	2–10
Eosinophils	2	%	0–6
Basophils	0.5	%	0–1
Platelet count	260	×10³/µL	150–400
Prothrombin time	12.4	Seconds	11–14
International normalized ratio	1.0	—	0.8–1.2
Activated partial thromboplastin time	30	Seconds	25–35
Fibrinogen	420	mg/dL	200–400
Blood urea nitrogen	10	mg/dL	7–20
Serum creatinine	0.6	mg/dL	0.5–1.1
Estimated glomerular filtration rate	>90	mL/minute/1.73 m²	>60
Aspartate aminotransferase	18	U/L	10–40
Alanine aminotransferase	20	U/L	7–56
Alkaline phosphatase	140	U/L	40–130*
Total bilirubin	0.6	mg/dL	0.2–1.2
Serum albumin	3.6	g/dL	3.5–5.0
Sodium	138	mmol/L	135–145
Potassium	4.1	mmol/L	3.5–5.0
Chloride	102	mmol/L	98–106
Bicarbonate	24	mmol/L	22–28
C-reactive protein	3	mg/L	<5
Erythrocyte sedimentation rate	18	mm/hour	<20
Protein	Negative	—	Negative
Glucose	Negative	—	Negative
Ketones	Negative	—	Negative
Red blood cells	0–1	/HPF	0–2
White blood cells	0–2	/HPF	0–5

**Figure 1:**
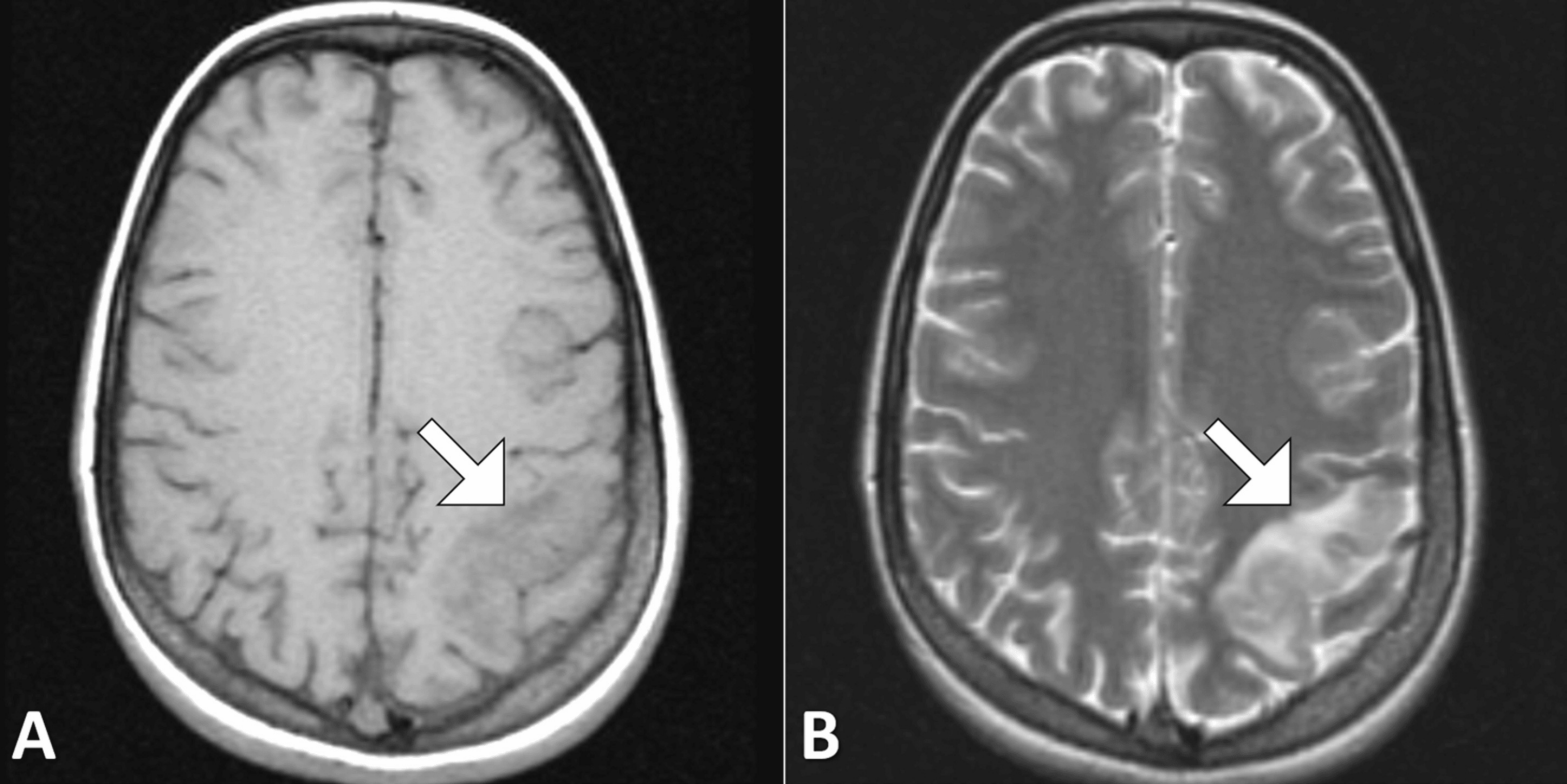
Axial T1- and T2-weighted MRI of the left parietal lobe. Axial T1-weighted MRI (A) shows a hypointense lesion (white arrow) in the left parietal cortex. Corresponding axial T2-weighted MRI (B) demonstrates hyperintensity at the same location (white arrow), mimicking a neoplastic lesion. T1 hypointensity and T2 hyperintensity without mass effect are suggestive but non-specific.

**Figure 2:**
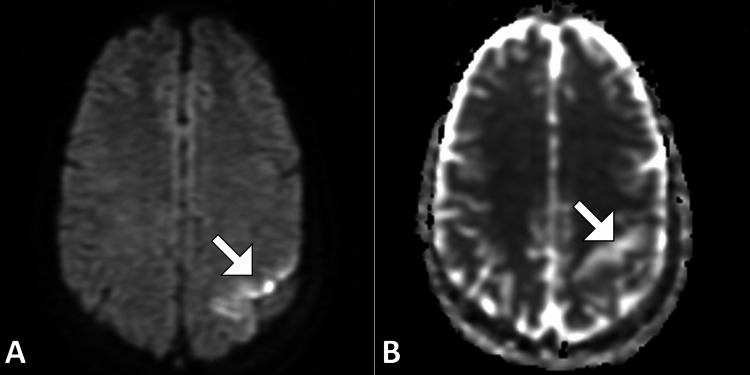
DWI and ADC mapping of the left parietal lobe. DWI (A) shows no areas of true restricted diffusion in the lesion. The corresponding ADC map (B) demonstrates facilitated diffusion in the same region (white arrow), consistent with vasogenic edema rather than cytotoxic edema. These findings help differentiate thrombotic edema from acute infarction. DWI = diffusion-weighted imaging; ADC = apparent diffusion coefficient

**Figure 3:**
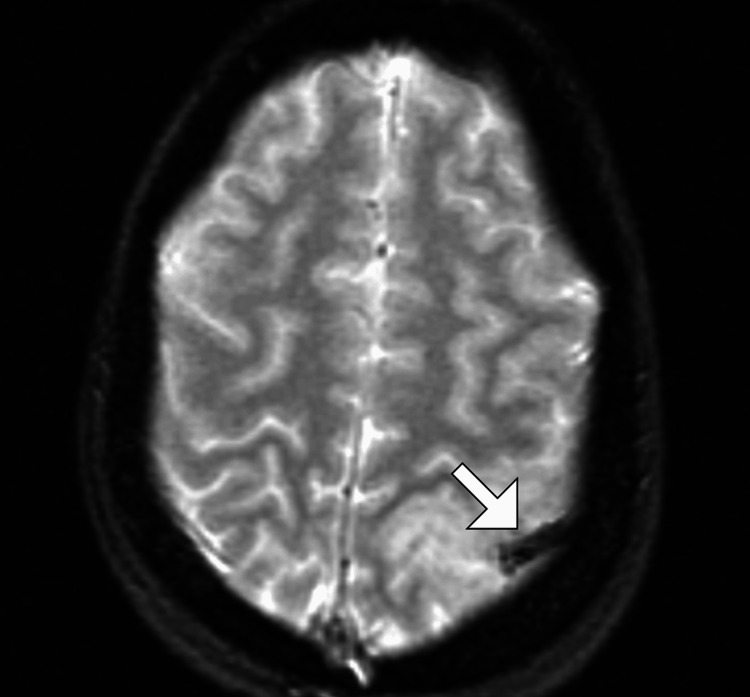
Gradient-echo MRI showing cortical vein thrombosis of the vein of Trolard. Axial gradient-echo MRI demonstrates a blooming artifact (white arrow) in a tubular structure corresponding to the thrombosed vein of Trolard. This blooming is characteristic of deoxygenated blood products within a thrombosed cortical vein and confirms the diagnosis of isolated cortical vein thrombosis.

MRI of the brain without contrast was performed. T1-weighted images demonstrated a poorly defined hypointense to isointense lesion in the left parietal lobe with mild mass effect on adjacent cortical sulci. T2-weighted and fluid-attenuated inversion recovery images revealed a hyperintense cortical-subcortical lesion with surrounding vasogenic edema, raising concern for a space-occupying lesion (Figure 1). Diffusion-weighted imaging showed areas of restricted diffusion within the cortical region, with corresponding low signal on the apparent diffusion coefficient map, suggestive of acute infarction (Figure 2). Gradient-echo sequences demonstrated a linear hypointense signal along the cortical surface consistent with a thrombosed cortical vein (Figure 3). Careful review identified involvement of the vein of Trolard, draining into the superior sagittal sinus, without evidence of sinus thrombosis. There was no significant midline shift or hemorrhagic transformation.

Based on the imaging appearance, the initial differential diagnosis included high-grade glioma, metastatic lesion, focal cerebritis, venous infarction secondary to CVT, and, less likely, tumefactive demyelination. The presence of restricted diffusion in a cortical distribution, associated venous signal abnormality on gradient-echo imaging, and the patient’s hypercoagulable state related to pregnancy favored a diagnosis of isolated CVT involving the vein of Trolard, presenting with venous infarction mimicking a tumor.

After a multidisciplinary discussion involving neurology, neurosurgery, radiology, obstetrics, and maternal-fetal medicine teams, a diagnosis of pregnancy-associated isolated CVT was established. Given the absence of intracranial hemorrhage and the progressive neurological deficits, anticoagulation therapy was initiated with therapeutic low-molecular-weight heparin at weight-adjusted dosing, which is considered safe in pregnancy. Dexamethasone was administered for a short course to reduce cerebral edema, and analgesics were given for pain control. Antiepileptic prophylaxis was not initiated as there was no seizure activity.

During her hospital course, the patient showed gradual neurological improvement. Headache intensity decreased significantly over the first 72 hours, and her speech and motor deficits improved to near baseline by day seven of hospitalization. Serial neurological examinations revealed no new deficits. Fetal monitoring remained reassuring throughout the admission, with no signs of fetal distress or preterm labor. A limited thrombophilia screen, deferred until the postpartum period due to pregnancy-related physiological changes, was planned.

She was discharged after 10 days of hospitalization on continued therapeutic low-molecular-weight heparin with close outpatient follow-up. At a six-week follow-up visit, she remained neurologically intact with complete resolution of focal deficits. Repeat MRI demonstrated a significant reduction in parietal edema and partial recanalization of the thrombosed cortical vein, with no evidence of a mass lesion. Anticoagulation was planned to be continued throughout pregnancy and the postpartum period, with coordinated follow-up by neurology and obstetrics.

## Discussion

CVT, particularly isolated CVT without concomitant dural sinus involvement, represents a rare and diagnostically challenging entity within the spectrum of cerebral venous thrombosis [1,4]. Its true incidence is likely underestimated due to heterogeneous clinical presentations and the frequent absence of pathognomonic imaging findings on routine neuroimaging [2,3]. The present case is notable for several reasons: the occurrence during pregnancy, isolated involvement of the vein of Trolard, and a radiological appearance closely mimicking an intracranial tumor. Together, these features highlight important diagnostic pitfalls and reinforce the need for heightened clinical and radiological awareness.

Pregnancy is a well-established prothrombotic state characterized by increased levels of clotting factors, reduced fibrinolytic activity, venous stasis, and endothelial dysfunction [2-4]. While cerebral venous sinus thrombosis is more commonly reported in the peripartum and postpartum periods, isolated CVT during pregnancy is exceptionally rare [3,5]. The absence of classic risk factors such as infection, dehydration, or known thrombophilia in this patient underscores the role of pregnancy alone as a sufficient precipitating factor [1-5].

Clinically, isolated CVT often presents with nonspecific symptoms such as headache and focal neurological deficits. Seizures are frequently reported, although their absence does not exclude the diagnosis [2,4]. The focal nature of symptoms reflects localized venous congestion and infarction, contrasting with the more diffuse manifestations seen in dural sinus thrombosis. The gradual progression of symptoms over several days may provide a subtle clinical clue favoring venous pathology over arterial stroke [3-7].

Radiologically, this case exemplifies the tumor-mimicking nature of CVT. Venous infarction may demonstrate cortical-subcortical edema, mass effect, and variable signal intensity on T1- and T2-weighted images [2,7]. Restricted diffusion further complicates differentiation, as both high-grade tumors and acute infarcts may demonstrate similar findings [2,7]. However, the cortical distribution of diffusion restriction and linear susceptibility signal on gradient-echo imaging corresponding to a thrombosed vein favors venous thrombosis [2,7]. Susceptibility-sensitive MRI sequences are therefore indispensable in avoiding misdiagnosis and unnecessary surgical intervention.

Management of isolated CVT parallels that of cerebral venous sinus thrombosis, with anticoagulation forming the cornerstone of therapy [1,3,8]. In pregnancy, low-molecular-weight heparin is the treatment of choice due to its safety profile. Early anticoagulation leads to excellent recovery and prevents thrombus propagation. Corticosteroids remain controversial but may be selectively used for significant vasogenic edema and mass effect [1,3,8].

## Conclusions

This case underscores the importance of considering isolated CVT as a critical differential diagnosis for tumor-like intracranial lesions in pregnant patients presenting with focal neurological deficits. Pregnancy-related hypercoagulability can predispose to rare venous thrombotic events involving cortical veins such as the vein of Trolard, with venous infarction closely mimicking neoplastic or inflammatory processes on neuroimaging. Careful interpretation of advanced MRI sequences is essential for accurate diagnosis. Early multidisciplinary evaluation and timely anticoagulation can result in excellent maternal and fetal outcomes.
